# Supplementary material for: Multispecies deep learning using citizen science data produces more informative plant community models
Source: Nat Commun. 2024 May 24;15:4421. doi: 10.1038/s41467-024-48559-9 (PMC11126635; doi:10.1038/s41467-024-48559-9)
Supplement: Supplementary file 1 — Supplementary Information [file 41467_2024_48559_MOESM1_ESM.pdf]

Supplementary Information

to the paper "Multispecies deep learning using citizen science data produces more informative plant community models" by Brun et al.

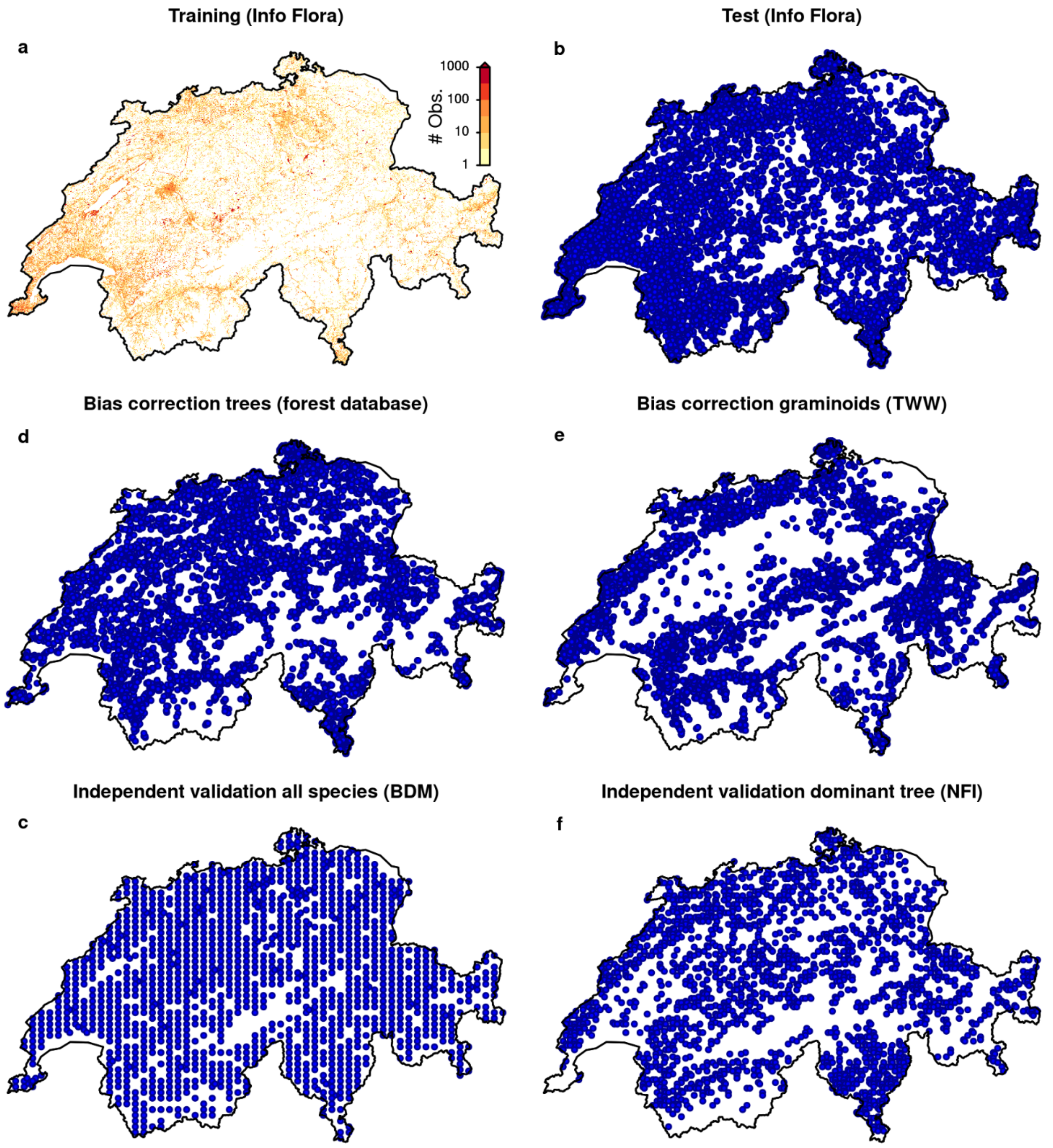

Supplementary Figure 1 | Spatial distributions of the observational data sets used in this study. a, number of quality-filtered training observations per 200×200 m pixel taken from the InfoFlora database. **b**, locations of quality-filtered left-out test observations taken from the InfoFlora database. **c**, locations of plant community observations for independent validation, taken from the Biodiversity Monitoring Program. **d**, locations of forest tree community observations for taxonomic bias correction, taken from the Swiss forest vegetation database. **e**, locations of grassland community plots for taxonomic bias correction, taken from the Dry Meadows and Pastures initiative. **f**, locations of forest tree community observations to validate model predictions taken from the National Forest Inventory. Maps were created using the open-source R software, with information on the country borders originating from the SwissTLM3D topographic landscape model (see methods).

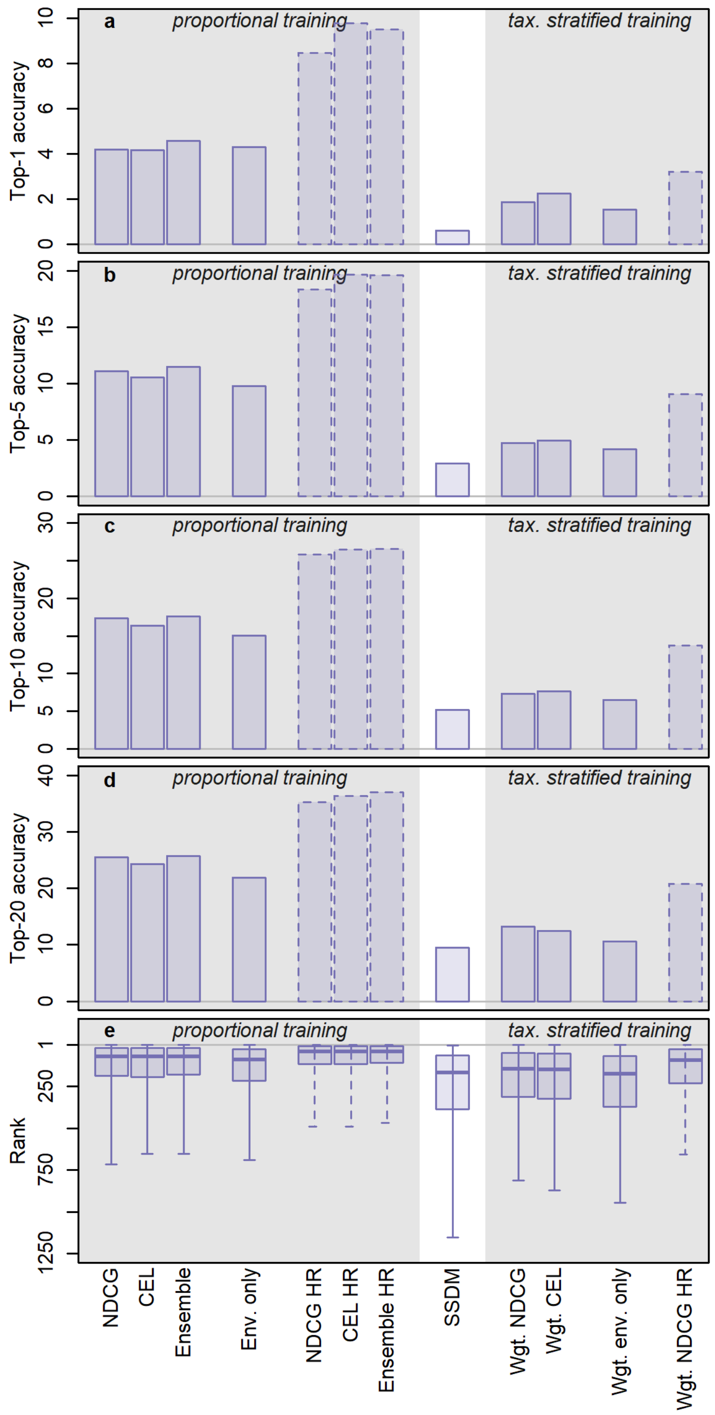

Supplementary Figure 2 | Overall performance of the trained deep neural networks and comparison to stacked species distribution models (SSDMs) against left-out test set of n=12'325 citizen science observations. Weighted versions were obtained by upsampling observations from rare taxa during training to obtain balanced taxon representation (see Methods). Top-1 accuracy in percent (**a**), top-5 accuracy in percent (**b**), top-10 accuracy in percent (**c**), top-20 accuracy in percent (**d**), and weighted ranks (**e**) are shown for different combinations of cost functions and predictor sets, as well as for predictions deduced from SSDMs. Note that we weighted the taxonomically balanced test set (five observations per species) with the number of training observations per species to obtain scores that represent typical field observations (see methods). Central lines in the boxplots of panel (**e**) indicate weighted medians, boxes indicate weighted interquartile ranges, and whiskers indicate weighted 2.5 and 97.5 percentiles. Env. represents environmental predictors only (i.e., no seasonal predictors); NDCG represents the normalized discounted cumulative gain cost function; Wgt. represents weighted; CEL represents the cross-entropy loss cost function; and HR represents high resolution. Source data are provided as a Source Data file.

Supplementary Table 1 | Predictive performance of the timing of blooming for all species with more than 10’000 training observations and 30 phenology observations. obs. represents observation; *r* refers to correlation coefficient. Source data are provided as a Source Data file.

Species	Validation obs. (#)	Training obs. (#)	Spearman *r*	Bias (days)
Achillea millefolium aggr.	71	19‘993	-0.01	-2
Ailanthus altissima	38	15‘029	-0.34	-33
Ajuga reptans	144	23‘381	0.62	4
Anemone nemorosa	495	11‘291	0.34	3
Anthoxanthum odoratum aggr.	110	39‘034	0.76	-0.5
Anthyllis vulneraria	70	15‘657	0.70	8
Arrhenatherum elatius	45	17‘411	0.50	5
Aster bellidiastrum	77	13‘021	0.59	4
Bellis perennis	310	16‘505	-0.11	45
Briza media	62	27‘213	0.71	-1
Bromus erectus	52	20‘907	0.16	4
Buddleja davidii	1639	44‘564	0.05	-17
Calluna vulgaris	68	17‘403	0.12	-26
Caltha palustris	206	21‘727	0.08	20
Carex flacca	37	26‘507	0.32	-1
Carlina acaulis	109	10‘033	-0.12	-27
Centaurea jacea aggr.	92	18‘438	0.23	-9
Colchicum autumnale	156	12‘549	0.17	-1.5
Corylus avellana	131	16‘023	-0.17	10
Dactylis glomerata	67	34‘116	0.55	5
Dactylorhiza maculata	7589	39‘479	0.46	0
Dactylorhiza majalis	2545	16‘766	0.81	11
Daucus carota	43	14‘312	0.42	-10
Epipactis atrorubens	2018	10‘703	0.52	-4
Epipactis helleborine aggr.	2617	15‘764	0.45	1
Erigeron annuus	4192	59‘858	0.26	16
Euphorbia cyparissias	35	12‘688	0.65	12
Filipendula ulmaria	70	22‘265	0.09	5.5
Galium mollugo aggr.	112	22‘811	0.41	8
Galium palustre aggr.	31	10‘467	0.27	1
Geranium robertianum	65	13‘405	0.64	4
Glechoma hederacea	179	12‘850	0.28	3
Gymnadenia conopsea	6585	27‘300	0.52	-3
Helianthemum nummularium	85	11‘703	0.22	-31
Heracleum mantegazzianum	65	18‘791	0.39	21
Heracleum sphondylium	58	16‘429	0.06	-22.5
Hieracium pilosella	31	10‘871	0.62	-62
Hippocrepis comosa	146	11‘766	0.43	9.5
Homogyne alpina	46	12‘986	0.52	19
Impatiens glandulifera	1463	39‘799	0.07	-1
Knautia arvensis	86	13‘450	0.32	11
Lamium galeobdolon	158	10‘389	0.48	7
Leontodon hispidus	77	24‘275	0.09	-41
Leucanthemum vulgare aggr.	119	27‘485	0.65	0

Listera ovata	3313	20‘431	0.76	1
Lotus corniculatus aggr.	240	39‘432	0.27	0
Medicago lupulina	93	15‘831	0.20	-8
Myosotis scorpioides aggr.	48	11‘956	0.48	8.5
Nardus stricta	35	17‘498	-0.05	-121
Neottia nidus-avis	2254	12‘195	0.37	-5
Nigritella rhellicani aggr.	4101	12‘113	0.38	1
Orchis mascula	4491	14‘384	0.52	8
Origanum vulgare	53	10‘541	-0.16	8
Oxalis acetosella	112	10‘602	0.48	3
Parnassia palustris	125	11‘306	0.04	-12
Phyteuma spicatum	54	11‘171	0.25	4
Plantago lanceolata	108	33‘291	0.29	0.5
Plantago media	52	16‘447	0.03	9
Platanthera bifolia	2324	10‘945	0.40	2
Polygonum bistorta	44	16‘541	0.03	23
Potentilla erecta	212	37‘758	0.31	0
Primula elatior	448	14‘267	0.34	12
Primula veris	496	10‘736	0.50	9
Prunella vulgaris	32	24‘731	0.11	-14
Prunus spinosa	37	10‘507	0.13	8
Ranunculus aconitifolius	64	11‘234	0.27	15.5
Ranunculus acris	109	31‘113	0.37	7
Ranunculus bulbosus	137	10‘051	0.41	9
Robinia pseudoacacia	69	19‘024	0.04	-115
Rosa canina aggr.	83	12‘273	0.25	-107
Rubus fruticosus aggr.	38	30‘543	0.30	-42.5
Rumex acetosa	51	20‘517	0.46	13
Salvia pratensis	131	12‘529	0.33	6
Sanguisorba minor	125	19‘712	0.24	3
Sanguisorba officinalis	40	12‘307	0.20	-5
Senecio inaequidens	897	157‘384	0.26	-36
Sesleria caerulea	78	10‘874	0.19	24.5
Silene flos-cuculi	41	10‘206	0.27	0
Solidago virgaurea	42	11‘448	0.34	-16
Succisa pratensis	110	12‘947	0.03	-2.5
Taraxacum officinale aggr.	161	30‘369	0.41	3
Thymus serpyllum aggr.	43	15‘146	0.30	-5

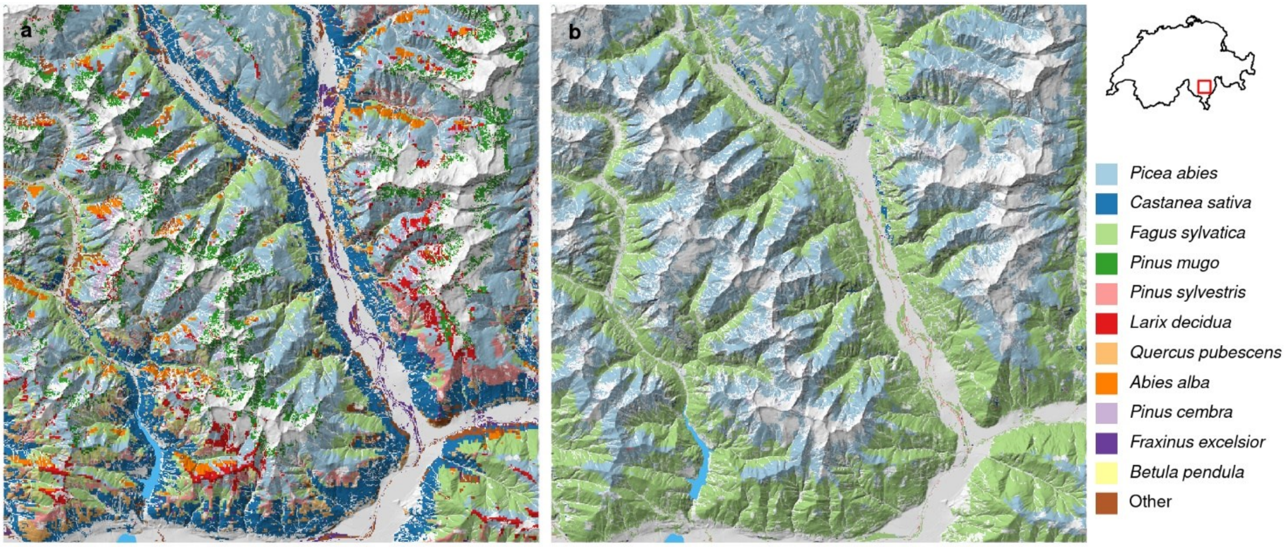

Supplementary Figure 3 | Potentially dominant canopy-forming tree species in wooded areas in northern Ticino (see inset map on the left). a, estimates according to reporting-bias corrected predictions of the low-resolution multispecies DNN with NDCG loss**; b,** estimates according to reporting-bias corrected predictions of stacked species distribution models**.** Colors represent species with the highest observation probability averaged from February to November (see legend). We compared observation probabilities of 37 tree species and tree species aggregates, as distinguished by the Swiss forest vegetation database, and masked pixels with land cover classes with no expected trees (see methods). Light blue represents *Picea abies*; dark blue represents *Castanea sativa*; light green represents *Fagus sylvatica*; dark green represents *Pinus mugo*; light red represents *Pinus sylvestris*; dark red represents *Larix decidua*; light orange represents *Quercus pubescens*; dark orange represents *Abies alba*; light purple represents *Pinus cembra*; dark purple represents *Fraxinus excelsior*; yellow represents *Betula pendula*; and brown represents other species. Maps were created using the open-source R software, with information on the country borders originating from the SwissTLM3D topographic landscape model (see methods).

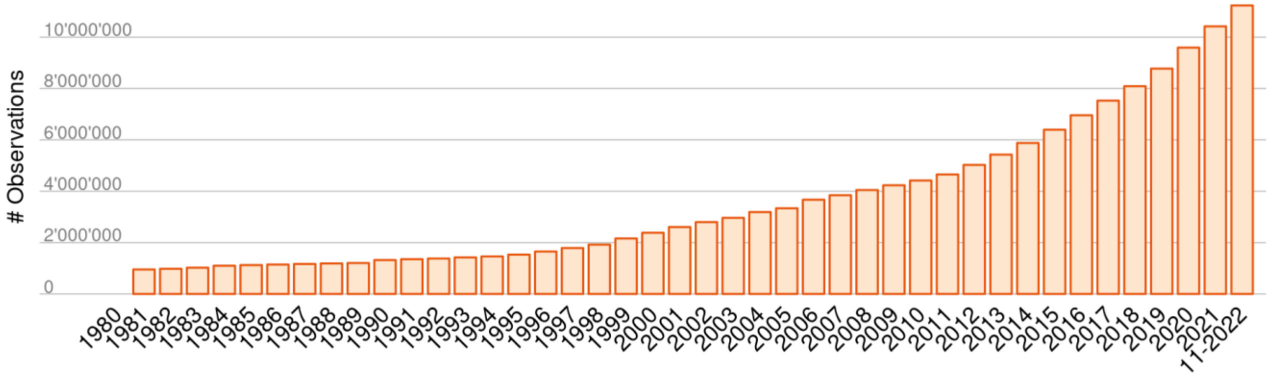

Supplementary Figure 4 | Increase of InfoFlora observations for the period 1980-2019. Depicted are raw data before the filtering steps mentioned in the methods. Source data are provided as a Source Data file.

	FCF	GDD_5	$Prec_{DJF}$	$Prec_{JJA}$	$Prec_{Annual}$	$SRad_{Annual}$	$Temp_{DJF}$	$Temp_{JJA}$	$Temp_{Annual}$	$NDVI_{Mean}$	$NDVI_{SD}$	Inner f. density	Canopy height	TPI	TRI	TWI	$Soil_D$	$Soil_F$	$Soil_H$	Continentality	Light availability	$Soil_N$	$Soil_R$
$Soil_W$	-0.35	0.7	0.09	0.09	0.01	0.46	0.66	0.69	0.69	0.54	0.24	0.29	0.32	-0.14	-0.54	0.51	-0.91	0.61	0.76	-0.36	-0.67	0.66	0.3
$Soil_R$	-0.3	0.56	0.03	-0.19	-0.25	0.41	0.55	0.56	0.57	0.17	0.25	0.15	0.17	-0.07	-0.25	0.24	-0.25	-0.09	-0.05	0.24	-0.32	0.5	
$Soil_N$	-0.58	0.91	-0.03	-0.18	-0.2	0.57	0.87	0.91	0.91	0.42	0.61	0.23	0.23	-0.11	-0.67	0.48	-0.75	0.26	0.42	-0.21	-0.62		
Light availability	0.34	-0.73	-0.09	-0.03	-0.01	-0.46	-0.66	-0.72	-0.71	-0.8	-0.21	-0.76	-0.79	0.09	0.25	-0.13	0.77	-0.16	-0.75	0.27			
Continentality	0.31	-0.1	-0.28	-0.37	-0.34	0.31	-0.04	-0.12	-0.09	-0.07	0.03	-0.23	-0.16	0.15	0.28	-0.18	0.31	-0.76	-0.3				
$Soil_H$	-0.16	0.5	0.13	0.2	0.15	0.39	0.45	0.48	0.47	0.82	0.22	0.41	0.45	-0.06	-0.26	0.16	-0.88	0.37					
$Soil_F$	-0.3	0.17	0.11	0.21	0.13	-0.17	0.13	0.18	0.16	-0.05	-0.02	0	-0.04	-0.16	-0.42	0.51	-0.45						
$Soil_D$	0.37	-0.78	-0.08	-0.05	0.01	-0.55	-0.74	-0.76	-0.76	-0.73	-0.4	-0.35	-0.38	0.12	0.56	-0.4							
TWI	-0.4	0.44	-0.16	-0.25	-0.26	0.27	0.47	0.44	0.45	-0.11	0.19	-0.1	-0.11	-0.31	-0.58								
TRI	0.4	-0.6	0.05	0.21	0.22	-0.44	-0.64	-0.59	-0.62	-0.08	-0.33	-0.02	0.05	0.07									
TPI	0.1	-0.09	0.05	0.06	0.06	0	-0.13	-0.08	-0.1	-0.03	-0.05	-0.02	-0.04										
Canopy height	-0.1	0.36	0.05	0.02	0.01	0.22	0.32	0.37	0.36	0.58	-0.09	0.92											
Inner f. density	-0.11	0.35	0.05	0.01	0.01	0.21	0.31	0.36	0.35	0.52	-0.08												
$NDVI_{SD}$	-0.36	0.48	-0.04	-0.15	-0.15	0.28	0.43	0.47	0.46	0.28													
$NDVI_{Mean}$	-0.11	0.52	0.12	0.11	0.09	0.44	0.47	0.5	0.5														
$Temp_{Annual}$	-0.57	0.99	-0.06	-0.18	-0.19	0.7	0.98	0.99															
$Temp_{JJA}$	-0.63	0.99	-0.08	-0.18	-0.2	0.65	0.94																
$Temp_{DJF}$	-0.45	0.95	-0.03	-0.19	-0.18	0.77																	
$SRad_{Annual}$	0	0.68	-0.06	-0.15	-0.14																		
$Prec_{Annual}$	0.32	-0.19	0.77	0.91																			
$Prec_{JJA}$	0.26	-0.17	0.59																				
$Prec_{DJF}$	0.26	-0.05																					
GDD_5	-0.61																						

Supplementary Figure 5 | Pearson correlation coefficients between low-resolution environmental predictors across Switzerland. Gray circles represent absolute Pearson correlation coefficients <0.7; orange circles represent absolute Pearson correlation coefficients between 0.7 and 0.9; and red circles represent absolute Pearson correlation coefficients higher than 0.9. $Soil_W$, $Soil_R$, $Soil_N$, $Soil_H$, $Soil_F$, and $Soil_D$, represent moisture variability, pH, nutrients, humus, moisture, and aeration, respectively; NDVI represents normalized difference vegetation index, Temp represents temperature, SRad represents solar radiation (direct and diffuse), Prec represents precipitation, GDD_5 represents growing degree days above five degrees Celsius, and FCF represents frost change frequency. SD represents standard deviation, DJF represents December, January, February, and JJA represents June, July, August. Source data are provided as a Source Data file.

	$Temp_{Annual}$	$Temp_{Range}$	$Prec_{Annual}$	$Prec_{JJA}$	$SRad_{Annual}$	$VegH_{Max}$	$VegH_{Q1}$	EVI_{Mean}	$EVI_{Seasonality}$	VMG	Aspect	TWI	TRI	$Soil_R$
$Soil_F$	-0.02	-0.13	0.31	0.37	-0.36	-0.04	0	0.02	0.26	-0.06	0.04	0.43	-0.41	-0.49
$Soil_R$	0.52	0.46	-0.4	-0.43	0.4	0.12	0.08	0.31	-0.26	0.18	0	0.06	0.11	
TRI	-0.32	-0.29	0.16	0.16	-0.11	0.24	0.12	-0.1	0.14	0.07	-0.01	-0.57		
TWI	0.4	0.4	-0.26	-0.26	0.18	-0.11	-0.06	0.13	-0.18	-0.02	0			
Aspect	0.04	0.05	0	0	-0.05	0.03	0.02	0.04	0.04	0				
VMG	0.27	0.21	-0.1	-0.14	0.15	0.46	0.5	0.41	0.07					
$EVI_{Seasonality}$	-0.27	-0.32	0.33	0.36	-0.25	0.07	0.03	-0.01						
EVI_{Mean}	0.66	0.5	-0.2	-0.24	0.31	0.55	0.41							
$VegH_{Q1}$	0.17	0.13	-0.06	-0.09	0.03	0.6								
$VegH_{Max}$	0.24	0.16	-0.04	-0.07	0.07									
$SRad_{Annual}$	0.48	0.51	-0.34	-0.33										
$Prec_{JJA}$	-0.38	-0.59	0.89											
$Prec_{Annual}$	-0.33	-0.64												
$Temp_{Range}$	0.74													

Supplementary Figure 6 | Pearson correlation coefficients between high-resolution environmental predictors across Switzerland. Gray circles represent absolute Pearson correlation coefficients <0.7; orange circles represent absolute Pearson correlation coefficients between 0.7 and 0.9. $Soil_R$ and $Soil_F$ represent pH and nutrients, and respectively; EVI represents enhanced vegetation index, Temp represents temperature, Prec represents precipitation, SRad represents solar radiation (direct and diffuse), VegH represents vegetation height, and VMG represents forest canopy mixture (deciduous versus evergreen). Q1 represents first quartile, Max represents maximum, and JJA represents June, July, August. Source data are provided as a Source Data file.

Supplementary Table 2 | Selection of low-resolution environmental predictors. “Times selected in SDMs“ refers to the species-specific variable selection procedure described in the subsection “Species distribution models” of the methods and is a proxy for variable importance; “Correlation cluster #” gives an imperfect indication of groups of predictors with absolute Pearson correlation coefficients > 0.7; “Selected” indicates the low-resolution predictors selected for DNNs (with “1”). $Soil_W$, $Soil_R$, $Soil_N$, $Soil_H$, $Soil_F$, and $Soil_D$, represent moisture variability, pH, nutrients, humus, moisture, and aeration, respectively; NDVI represents normalized difference vegetation index, Temp represents temperature, SRad represents solar radiation (direct and diffuse), Prec represents precipitation, GDD_5 represents growing degree days above five degrees Celsius, and FCF represents frost change frequency. SD represents standard deviation, DJF represents December, January, February, and JJA represents June, July, August. Source data are provided as a Source Data file.

Name	Times selected in SDMs	Correlation cluster #	Selected
TRI	2224	10	1
$Soil_R$	2103	13	1
$NDVI_{SD}$	1794	7	1
$NDVI_{Mean}$	1602	6	1
$SRad_{Annual}$	1582	5	0
Canopy height	1577	8	1
$Prec_{JJA}$	1496	4	1
FCF	1352	1	1
$Prec_{DJF}$	1313	3	1
$Soil_F$	1276	12	1
TPI	1185	9	0
$Soil_N$	1117	2	0
$Soil_W$	1056	2	0
Continentality	999	12	0
TWI	948	11	0
Inner forest density	642	8	0
$Temp_{JJA}$	628	2	1
GDD_5	524	2	0
$Soil_D$	524	2	0
$Soil_H$	501	6	0
$Prec_{Annual}$	438	3	0
Light availability	412	6	0
$Temp_{Annual}$	411	2	0
$Temp_{DJF}$	365	2	0

Supplementary Table 3 | P-values of paired, two-sided Wilcoxon tests. Source data are provided as a Source Data file.

**Metric**	**Group 1**	**Group2**	***n***	**Statistic**	***p***	***p* adjusted**	
Rank	CEL	Ensemble	12325	39043560	3.47E-65	6.94E-65	****
Rank	CEL	NDCG	12325	34123132	3.27E-01	3.27E-01	ns
Rank	CEL	SDM	12325	12203025	0	0	****
Rank	Ensemble	NDCG	12325	23136256	1.01E-117	3.03E-117	****
Rank	Ensemble	SDM	12325	11136834	0	0	****
Rank	NDCG	SDM	12325	13031791	0	0	****
AUC by species	CEL	Ensemble	1489	1044085	0.006	0.012	*
AUC by species	CEL	NDCG	1489	1125435	0.472	0.472	ns
AUC by species	CEL	SDM	1489	1415340	4.5E-39	2.25E-38	****
AUC by species	Ensemble	NDCG	1489	1191508	0.000407	0.001	**
AUC by species	Ensemble	SDM	1489	1473774	1.22E-54	7.32E-54	****
AUC by species	NDCG	SDM	1489	1407026	4.47E-37	1.79E-36	****
AUC by site	CEL	Ensemble	1345	277661.5	3.7E-27	1.85E-26	****
AUC by site	CEL	NDCG	1345	508994.5	1.14E-09	3.42E-09	****
AUC by site	CEL	SDM	1345	480371	0.002	0.004	**
AUC by site	Ensemble	NDCG	1345	687439	6.94E-87	4.16E-86	****
AUC by site	Ensemble	SDM	1345	522667	5.41E-10	2.16E-09	****
AUC by site	NDCG	SDM	1345	417837	0.176	0.176	ns

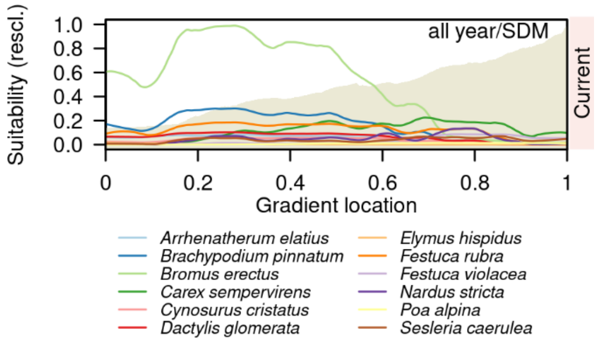

Supplementary Figure 7 | Reporting bias-corrected observation probabilities of graminoid taxa along an elevational gradient in the canton of Vaud (see Fig. 4 for mapped location). All-year habitat suitability under current conditions as derived from reporting-bias corrected predictions of stacked species distribution models. Yellowish shading in the background represents the elevation profile ranging from 400 to 2900 m asl. Illustrated are the twelve taxa with the most frequent potential dominance according to multispecies DNNs. Light blue represents *Arrhenatherum elatius*; dark blue represents *Brachypodium pinnatum*; light green represents *Bromus erectus*; dark green represents *Carex sempervirens*; light red represents *Cynosurus cristatus*; dark red represents *Dactylis glomerata*; light orange represents *Elymus hispidus*; dark orange represents *Festuca rubra*; light purple represents *Festuca violacea*; dark purple represents *Nardus stricta*; yellow represents *Poa alpina*; and brown represents *Sesleria caerulea*. Source data are provided as a Source Data file.

Supplementary Note 1: Identifying optimal number of training epochs

We conducted preliminary training runs to identify the optimal number of training epochs for models with unweighted sampling probabilities. To do so, we trained the low-resolution models with environmental and seasonal predictors, once with the cross-entropy loss (CEL) and once with the normalized discounted cumulative gain (NDCG) as cost functions. In both cases, we used the validation data set to quantify the test statistic, i.e., top-1 accuracy, after each epoch, and the remaining data for training. Both validation runs were run for 500 epochs with the same settings as used for the main runs (see methods). Based on the resulting curves of top-1 accuracy (Supplementary Fig. 8), we selected 300 epochs as training duration for the CEL cost function and 200 epochs as training duration for the NDCG cost function.

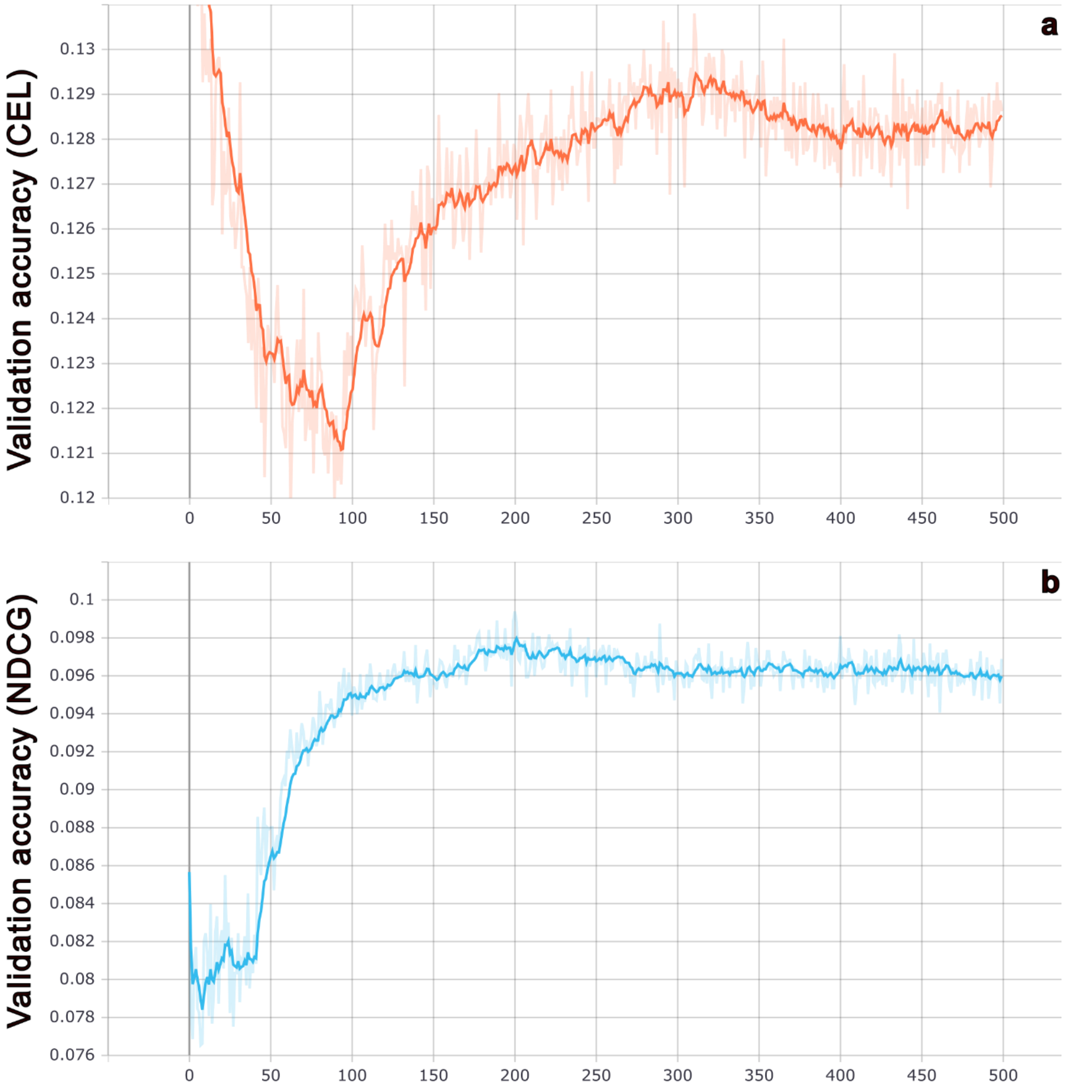

Supplementary Figure 8 | Evolution of validation scores for low-resolution DNNs trained with all predictors. **a**, validation accuracy for the low-resolution CEL DNN tracked over 500 epochs. **b**, validation accuracy for the low-resolution NDCG DNN tracked over 500 epochs. Note that prior to this validation run, both models had been trained with taxonomically stratified sampling probabilities for 50 epochs (leading to high accuracy scores in the taxonomically stratified test set).
