# Supplementary material for: Multispecies deep learning using citizen science data produces more informative plant community models
Source: Nat Commun. 2024 May 24;15:4421. doi: 10.1038/s41467-024-48559-9 (PMC11126635; doi:10.1038/s41467-024-48559-9)
Supplement: Supplementary file 3 — Reporting Summary [file 41467_2024_48559_MOESM3_ESM.pdf]

nature portfolio

Corresponding author(s): Philipp Brun

Last updated by author(s): Jan 16, 2024

Reporting Summary

Nature Portfolio wishes to improve the reproducibility of the work that we publish. This form provides structure for consistency and transparency in reporting. For further information on Nature Portfolio policies, see our Editorial Policies and the Editorial Policy Checklist.

Statistics

For all statistical analyses, confirm that the following items are present in the figure legend, table legend, main text, or Methods section.

n/a	Confirmed	
☐	☒	The exact sample size (*n*) for each experimental group/condition, given as a discrete number and unit of measurement
☒	☐	A statement on whether measurements were taken from distinct samples or whether the same sample was measured repeatedly
☐	☒	The statistical test(s) used AND whether they are one- or two-sided<br>*Only common tests should be described solely by name; describe more complex techniques in the Methods section.*
☐	☒	A description of all covariates tested
☐	☒	A description of any assumptions or corrections, such as tests of normality and adjustment for multiple comparisons
☐	☒	A full description of the statistical parameters including central tendency (e.g. means) or other basic estimates (e.g. regression coefficient) AND variation (e.g. standard deviation) or associated estimates of uncertainty (e.g. confidence intervals)
☐	☒	For null hypothesis testing, the test statistic (e.g. *F*, *t*, *r*) with confidence intervals, effect sizes, degrees of freedom and *P* value noted<br>*Give P values as exact values whenever suitable.*
☒	☐	For Bayesian analysis, information on the choice of priors and Markov chain Monte Carlo settings
☒	☐	For hierarchical and complex designs, identification of the appropriate level for tests and full reporting of outcomes
☐	☒	Estimates of effect sizes (e.g. Cohen's *d*, Pearson's *r*), indicating how they were calculated

Our web collection on statistics for biologists contains articles on many of the points above.

Software and code

Policy information about availability of computer code

Data collection	Data was mostly available/provided in tabular form or as geoTIFFS and without the need of software for collection. Geotiffs were compiled with the in R (version 4.0.4) using the package terra (terra_1.7-71). However, we used SAGA GIS to derive nationwide maps of aspect and SAGA wetness index from the SwissAlti3D digital elevation model. For InfoFlora, the main origin of observational data, most observations were submitted to the database via smartphone, using the app "FlorApp". We accessed Info Flora data through an API, using R (version 4.0.4) and the rjson R-package (version 0.2.20).
Data analysis	Data preparation, species distribution modelling, and analysis and visualization of model outputs were conducted in the R environment (versions 4.0.4 and 3.6.3 for preparation/SDMs, and output analysis/visualization, respectively), using the packages terra (version 1.7-71), mgcv (version 1.8-42), randomForest (version 4.6-14), gbm (version 2.1.8), dismo (version 1.3-2), ROCR (version 1.0-11), rstatix (version 0.7.2), reticulate (version 1.30), RColorBrewer (version 1.1-3), and magick (version 2.7.4). DNNs were fitted in python (version 3.8.5) using the PyTorch library (version 1.7.1), the pytorchltr library (version 0.2.1), the pandas library (version 1.1.3), and the numpy library (version 1.19.2). Code underlying the main analyses conducted in this study, as well as minimal data sets to run it are available on GitHub (https://doi.org/10.5281/zenodo.10869585).

For manuscripts utilizing custom algorithms or software that are central to the research but not yet described in published literature, software must be made available to editors and reviewers. We strongly encourage code deposition in a community repository (e.g. GitHub). See the Nature Portfolio guidelines for submitting code & software for further information.

Data

Policy information about availability of data

All manuscripts must include a data availability statement. This statement should provide the following information, where applicable:

- Accession codes, unique identifiers, or web links for publicly available datasets
- A description of any restrictions on data availability
- For clinical datasets or third party data, please ensure that the statement adheres to our policy

Data sets used in this study include observational data and environmental data. The observational data from InfoFlora can be obtained for scientific research via online request under https://www.infoflora.ch/en/data/request-data.html. The accuracy of the spatial coordinates provided may be restricted depending on the context of the project and the confidentiality of the individual data. A standard extraction fee of CHF 175 may apply to user groups other than public research institutions. The observational data from the National Forest Inventory can be obtained after signing a written agreement, with contact details provided under https://www.lfi.ch/dienstleist/daten-en.php?lang=en. An extraction fee may apply to user groups other than public research institutions. The observational data from the Swiss Biodiversity Monitoring Program can be obtained after signing a written agreement with contact details provided under https://biodiversitymonitoring.ch/index.php/en/service/data-orders. Finally, the observational data from the Swiss forest vegetation database are available upon request as described under https://www.givd.info/ID/EU-CH-005. For environmental data, the LiDAR-based estimates of canopy height and inner forest density are available upon request as described under https://www.doi.org/10.16904/envidat.148; Landsat measurements of the normalized difference vegetation index (NDVI) are available under https://espa.cr.usgs.gov/; data from the EarthEnv-DEM90 digital elevation model can be downloaded und https://www.earthenv.org/DEM; reanalysis data on climate variables are available from https://doi.org/10.24381/cds.adbb2d47; plant indicator value-based estimates of soil properties, continentality, and light availability are available under https://www.doi.org/10.16904/envidat.153; annual and seasonal averages of high-resolution measurements of the enhanced vegetation index (EVI) for Switzerland are available under https://doi.org/10.26037/yareta:hapbjzl6dvbwnb5modewqozbfm, https://doi.org/10.26037/yareta:tilf3ibfnrafjpj6xpnea3vhpm, https://doi.org/10.26037/yareta:of5ddowrxvbtjjurioduueopey, and https://doi.org/10.26037/yareta:hgw56omleveiplgftnd5ugwpja, for 2018, 2019, 2020, and 2021, respectively; high-resolution data on the vegetation height of Switzerland are available upon request as described under https://www.doi.org/10.16904/1000001.1; high-resolution data on forest type in Switzerland are available upon request as described under https://www.doi.org/10.16904/1000001.7; data on temperature and precipitation from the CHclim25 data set are available under https://doi.org/10.5281/zenodo.10635681; data on land use/land cover are available under https://doi.org/10.26037/yareta:dlx3hu54jfa3ne3c2xjfcnqpxm; high-resolution elevation data for Switzerland from the SwissAlti3D digital elevation model are available under https://www.swisstopo.admin.ch/en/height-model-swissalti3d; and high-resolution data on rivers and lakes from the SwissTLM3D topographic landscape model are available under https://www.swisstopo.admin.ch/en/landscape-model-swisstlm3d.

Research involving human participants, their data, or biological material

Policy information about studies with human participants or human data. See also policy information about sex, gender (identity/presentation), and sexual orientation and race, ethnicity and racism.

Reporting on sex and gender	Does not apply
Reporting on race, ethnicity, or other socially relevant groupings	Does not apply
Population characteristics	Does not apply
Recruitment	Does not apply
Ethics oversight	Does not apply

Note that full information on the approval of the study protocol must also be provided in the manuscript.

Field-specific reporting

Please select the one below that is the best fit for your research. If you are not sure, read the appropriate sections before making your selection.

☐ Life sciences ☐ Behavioural & social sciences ☒ Ecological, evolutionary & environmental sciences

For a reference copy of the document with all sections, see nature.com/documents/nr-reporting-summary-flat.pdf

Ecological, evolutionary & environmental sciences study design

All studies must disclose on these points even when the disclosure is negative.

Study description	We jointly modelled the distribution of a major part of the Swiss flora, using deep neural networks (DNNs) with different cost functions and compared the results to predictions by an ensemble of species distribution models. The response variable was conditional observation probability for each species and species aggregate (multiclassification) in the case of DNNs and habitat suitability in the case of species distribution models. Models were based on >5 Mio species observations in total. Environmental predictors (10-20) represented vegetation structure, climate, soil conditions, topography, and season.
Research sample	We modeled the distribution of 2477 vascular plant species and species aggregates within Switzerland.<br>Data originated from several national databases, including sources that were mostly citizen science based (Info Flora: 6.7 Mio species observations) and sources that were expert based (the Swiss biodiversity monitoring program: 1489 survey sites, the national forest

	inventory: 1867 survey sites, the Swiss forest vegetation database: 12'911 survey sites, and the dry meadows and pastures initiative: 23'919 survey sites).
Sampling strategy	We expected a minimum sample size of 8 spatially thinned observations for species distribution modelling (using ensembles of small models) and a minimum sample size of 20 training and 5 test observations for DNNs. We only considered taxa that fulfilled both criteria for the comparative analyses.
Data collection	Data originated from several national databases, including sources that were mostly citizen science based (Info Flora) and sources that were expert based (the Swiss biodiversity monitoring program, the national forest inventory, the Swiss forest vegetation database, and the dry meadows and pastures initiative).
Timing and spatial scale	Observations made in 1971 or more recently were considered, with 80% of Info Flora observations and the majority of expert-based observations made after 1998. Spatial extent is Switzerland; resolution is 25x25 and 100x100 m. See Methods, and Extended Data Figs 1 & 5 for more details.
Data exclusions	Observations were excluded<br>- if they were made outside of Switzerland<br>- if coordinate uncertainty was too high (see Methods for details)<br>- if identifications were made at genus level or cruder, or of species with too few observations to be considered for modeling<br>- if identifications were marked as uncertain by citizen scientists or flagged as doubtful by Info Flora
Reproducibility	No experiments were conducted. Code will be made available on Gitlab after acceptance of the MS.
Randomization	Five observations per taxon were sampled for the citizen sciene-based test data. Sampling was made randomly under the constraint that observations had be made in April 2019 or more recently, wherever possible. This was necessary to have the same test set for high-resolution and low-resolution analyses (see Methods for details). Otherwise, no randomizations were made.
Blinding	Blinding was not relevant to this study.

Did the study involve field work? ☐ Yes ☒ No

Reporting for specific materials, systems and methods

We require information from authors about some types of materials, experimental systems and methods used in many studies. Here, indicate whether each material, system or method listed is relevant to your study. If you are not sure if a list item applies to your research, read the appropriate section before selecting a response.

Materials & experimental systems

n/a	Involved in the study
☒	☐ Antibodies
☒	☐ Eukaryotic cell lines
☒	☐ Palaeontology and archaeology
☒	☐ Animals and other organisms
☒	☐ Clinical data
☒	☐ Dual use research of concern
☒	☐ Plants

Methods

n/a	Involved in the study
☒	☐ ChIP-seq
☒	☐ Flow cytometry
☒	☐ MRI-based neuroimaging
